# Molecular response assessment using circulating tumor DNA (ctDNA) in advanced solid tumors

**DOI:** 10.1038/s41416-023-02445-1

**Published:** 2023-10-03

**Authors:** Jeffrey C. Thompson, Dylan G. Scholes, Erica L. Carpenter, Charu Aggarwal

**Affiliations:** 1 Division of Pulmonary, Allergy and Critical Care Medicine, Thoracic Oncology Group, Department of Medicine, Perelman School of Medicine, University of Pennsylvania, Philadelphia, PA USA; 2 Abramson Cancer Center, Philadelphia, PA USA; 3 Division of Hematology Oncology, Department of Medicine, Perelman School of Medicine, University of Pennsylvania, Philadelphia, PA USA; 4 Penn Center for Cancer Care Innovation, Philadelphia, PA USA

**Keywords:** Tumour biomarkers, Non-small-cell lung cancer

## Abstract

The therapeutic landscape for patients with advanced malignancies has changed dramatically over the last twenty years. The growing number of targeted therapies and immunotherapeutic options available have improved response rates and survival for a subset of patients, however determining which patients will experience clinical benefit from these therapies in order to avoid potential toxicities and reduce healthcare costs remains a clinical challenge. Cell-free circulating tumor DNA (ctDNA) is shed by tumor cells into systemic circulation and is already an integral part of routine clinical practice for the non-invasive tumor genotyping in advanced non-small cell lung cancer as well as other malignancies. The short half-life of ctDNA offers a unique opportunity to utilize early on-treatment changes in ctDNA for real-time assessment of therapeutic response and outcome, termed *molecular response*. Here, we provide a summary and review of the use of molecular response for the prediction of outcomes in patients with advanced cancer, including the current state of science, its application in clinic, and next steps for the development of this predictive tool.

## Introduction

Based upon an improved understanding of the biology of cancer, we have witnessed a paradigm change in the management of patients with advanced malignancies. Treatment options including targeted therapies for oncogene-addicted tumors and checkpoint inhibitors have contributed to significant improvements in quality of life, toxicity and overall survival outcomes for patients. Despite these improvements, responses are often limited to a subset of patients, and additionally, the efficacy of targeted therapies is limited by the emergence of resistant populations of tumor cells [1, 2]. Determining which patients will experience clinical benefit from these therapies remains a clinical challenge. Early and accurate prediction of response would allow patients deriving clinical benefit to continue therapy, while avoiding unnecessary toxicities and enabling re-stratification to more effective therapies for those unlikely to respond. Disease monitoring utilizing clinical assessment and computed tomography (CT) imaging is the current standard of care to assess response to therapy. However, radiographic evaluation does not fully represent the molecular and pathologic changes that occur within the tumor microenvironment during treatment and clinical benefit to these therapies may extend beyond conventional radiologic assessment of tumor response [3, 4]. Repeat tumor biopsies are often infeasible, are invasive with the potential for complications, and may not fully capture the complexity of intra and inter-tumor heterogeneity. As a result, there is a significant clinical need to develop non-invasive, early on-treatment approaches to accurately identify the subgroup of patients most likely to respond to these therapies to facilitate real-time personalized treatment decisions.

Cell-free circulating tumor DNA (ctDNA) is shed by tumor cells into systemic circulation. With advancements in sequencing technologies offering the ability to detect mutations, plasma genotyping of ctDNA is increasingly being utilized to identify driver mutations and match patients to the appropriate personalized therapies. The use of ctDNA offers the advantage of being minimally invasive, can be repeated sequentially, and may provide a more systemic view of tumor clonal evolution over time [5]. Plasma genotyping using ctDNA is already an integral component of routine clinical practice for a variety of tumor types. In the setting of certain advanced malignancies, such as non-small cell lung cancer (NSCLC), ctDNA is predominantly utilized as a complement to tissue genotyping to assist in selection of initial therapy at diagnosis, and is being employed as a real-time tool for monitoring of emergent resistance mutations in patients receiving targeted therapy. In addition to these applications, the short half-life of ctDNA offers a unique opportunity to utilize early on-treatment changes in ctDNA for real-time assessment of therapeutic response and outcome, termed *molecular response*. The use of molecular response is a promising approach to guide therapeutic decisions early in the course of treatment and as an emerging biomarker with broad implications for integration in the clinic and design of clinical trials. Here we provide a summary and review of molecular response for the prediction of outcomes in patients with advanced cancer, including the current state of science, its application in clinic, and next steps for the development of this predictive tool.

## Methods of ctDNA analysis for measuring molecular response

Plasma cell-free DNA (cfDNA) consists of short DNA fragments (about 150–200 base pairs) secreted or released into the bloodstream through apoptosis or necrosis with a half-life of approximately one to two hours in circulation [6]. The majority of cfDNA is derived from normal cells, but in individuals with cancer, tumor cells may also release cfDNA that is specifically termed ctDNA. ctDNA often represents a small proportion of the total cfDNA (median around 0.4%) [7], thus, highly sensitive and specific sequencing methods are needed to detect rare somatic mutations and copy-number changes. Analytic methodologies for ctDNA detection and analysis range from limited polymerase chain reaction (PCR)-based approaches to broader coverage next-generation sequencing (NGS) platforms. Detailed review of specific plasma genotyping methodologies is beyond the scope of this article and has been reviewed elsewhere [8, 9]. The most common ctDNA analysis methods utilized in studies assessing molecular response are droplet digital PCR (ddPCR) and next generation sequencing (NGS). ddPCR is highly sensitive, inexpensive, and has a rapid turnaround time (~2–3 days). PCR-based approaches interrogate a limited number of known mutations with high sensitivity and specificity and can be used to track predefined mutations of interest over time. NGS, or high throughput sequencing or massive parallel sequencing, technologies offer the advantage of simultaneously sequencing a large panel of genes up to the whole exome in a single run, which allows for tracking a more complete picture of the tumor over time. While NGS assays provide a broad spectrum of genomic information, they can sometimes have limited sensitivity, compared to PCR assays for specific single nucleotide variants [10]. The most common readout for a mutation detected in ctDNA is the variant allele frequency (VAF), defined as the fraction of cfDNA molecules sequenced at a particular locus that carry the specific variant. Less commonly, the amount of plasma containing a particular variant may also be described by the ctDNA concentration of mutant molecules per volume of plasma, i.e. milliliter.

## Methods of calculating molecular response

Assessment of molecular response involves the measurement of ctDNA kinetics between baseline ctDNA VAF and a prespecified early on-treatment timepoint to identify therapeutic response and predict patient outcomes (Table 1). While the optimal timing of the first on-treatment timepoint is currently under investigation, various studies have analyzed intervals varying between 2–12 weeks from baseline [11–20].

**Table 1:** Serial ctDNA Testing Categories.

	Molecular response	Longitudinal monitoring	At-progression testing
Definition	Assess changes in ctDNA in patients on treatment (~3–9 wks) vs. baseline	Assess increases and/or decreases in ctDNA over time	Use of ctDNA testing with clinical evidence of progressive disease
Primary Use	Predict eventual outcomes/response to current therapy	Monitoring (correlation with patient disease status)	Interrogation of emergent resistance mutations, increase in VAF to correlate with clinical evidence of progressive disease
Clinical Trigger	Start of initial or new therapy	Regular cadence (i.e. q3 months) OR when additional information needed to make clinical decision	Clinical signs of progression

*ctDNA* cell-free circulating tumor DNA, *VAF* variant allele frequency, *wks* weeks.

Various methods of measuring ctDNA molecular response have been published and can be grouped into three main categories: 1) ctDNA clearance, 2) delta VAF, and 3) proportion or ratio VAF methods. Clearance of ctDNA is a simple binary assessment of the presence or absence of detectable ctDNA at the early on-treatment timepoint in patients with detectable ctDNA at baseline. It may refer to the reduction in all baseline somatic variants below the limit of detection at an early on-treatment timepoint, or in the setting of receipt of targeted therapy, will refer to the clearance of the specific driver mutation(s) being targeted (e.g. *EGFR* L858R or *EML4-ALK* fusion). Measurement of ctDNA clearance is easy to calculate, and has been shown to effectively predict outcomes in a number of studies across multiple tumor types including NSCLC, breast cancer, and others [14, 21, 22]. This methodology, however, does not take in account patients that may not have complete clearance, but may still have a meaningful decrement in ctDNA with minimal, but persistent detectable ctDNA present while on treatment.

**Fig. 1:**
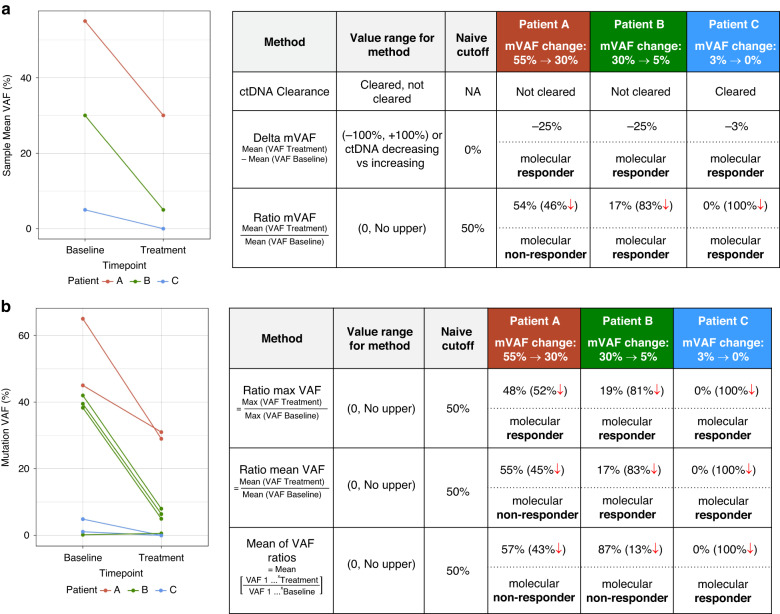
Impact of various molecular response calculations on patient results. When testing three different mean VAF (mVAF) examples, using three commonly published MR methods, ctDNA clearance, Delta mVAF, and ratio mVAF, can provide different MR results for the same case (1A). For example, patient A did not clear ctDNA on treatment and had only a 46% decrease by the ratio mVAF method, both of which are considered molecular non-response by those methods; but the same patient had a delta in mVAF of -25% which is considered molecular response by that method. Similarly variations of the ratio mVAF calculation can produce differing MR results for the same patient (1B). For example, Patient A is considered a molecular responder using the ratio max VAF, but a molecular non-responder using the ratio mVAF and mean of VAF ratios calculation.

Delta VAF (dVAF), which is measured as an increase or decrease in ctDNA VAF between baseline and an on-treatment timepoint has also been used, and demonstrated utility across several studies as a predictor of outcomes [23–26]. There is however considerable variation in the calculation based on whether all mutations at baseline are included, or only a single mutation. For example, some studies calculate delta VAF (dVAF) by subtracting the mean baseline VAF for all mutations from the mean on-treatment VAF [23–25]. Using this method, a decrease in dVAF has been correlated with statistically significant improvements in progression free survival (PFS), overall survival (OS), and/or objective response rates compared to an increase in dVAF ctDNA levels [17, 23, 24, 26]. The disadvantage of this approach is that it only assesses the relative change in ctDNA mean VAF over time and does not account for the residual ctDNA on-treatment [17]. For example, using this method, the molecular response of a patient who exhibits a decrease from a 50% mean VAF at baseline to 40% VAF on-treatment will be regarded the same as a patient whose ctDNA decreases from 10% to 0% (Fig. 1a), which may be misleading and not truly representative of the degree of response as ctDNA levels tend to correlate with overall tumor burden [27, 28].

The proportional change, percent change, ratio VAF methods can help account for both the relative change in ctDNA as well as the amount of residual ctDNA remaining at the on-treatment timepoint [16–18, 29, 30]. While studies employing this methodology all involve assessing on-treatment ctDNA relative to baseline ctDNA, there are nuanced differences to the formulas used in these studies, with a variety of inputs including mean VAF versus maximum VAF and the ratio of the mean VAF versus mean of individual alteration VAF ratios (Fig. 1b). Incorporation of mean rather than maximum VAF reduces the impact of possible stochastic sampling bias, temporal heterogeneity in VAF measurements, the max VAF alteration occurring on an amplified gene, and the potential alterations stemming from clonal hematopoiesis [31]. Studies by both Zhang et al. and Thompson et al. have compared the ratio mean VAF to other methods such as ctDNA clearance, delta mean VAF and found the ratio mean VAF algorithm to perform best for predicting overall response, PFS, and OS [17, 18]. However, future prospective trials are needed to determine the optimal method for calculating ctDNA molecular response.

## Applications of molecular response in advanced solid tumors

### Molecular response in NSCLC

The utilization of molecular response to predict clinical outcomes has been most widely studied in patients with advanced NSCLC, likely owing to the large number of recent FDA-approved and investigational therapies (Table 2). Initial analyses focused on targeted therapies, including a study by Marchetti et al., demonstrating that early reductions in ctDNA during treatment with erlotinib correlated with radiographic response [32]. Other studies have shown an association between early clearance of ctDNA analyzed between 2-8 weeks and PFS in patients with *EGFR* mutant NSCLC in trials of gefitinib, afatinib, osimertinib, and multiple other EGFR TKIs [14, 26, 33–37]. The largest of these trials utilized ddPCR to analyze baseline and on-treatment samples from 489 patients in the FLAURA trial, a first-line trial of osimertinib versus comparator EGFR TKI in patients with *EGFR* mutant NSCLC. In this study, patients with clearance of the respective *EGFR* exon 19 deletion or L858R mutation at three weeks on therapy had a longer PFS than those without clearance of the *EGFR* mutation (19.8 vs 11.3 months and 10.8 vs 7.0 months for those treated with osimertinib and comparator EGFR TKI, respectively. Similar results were seen with clearance of ctDNA at week six [14].

**Table 2:** Representative molecular response studies.

Author	Pub year	Cancer type	Treatment	ctDNA method	On-treatment ctDNA Time Point	PFS		OS		
**Targeted therapy**										
Kwon [38]	2022	NSCLC	Targeted therapy	NGS	2 months	Median PFS	-	Median OS		-
						HR	0.29 (0.13–0.66)	HR		0.23 (0.06–0.8)
						*P*-value	*p* = 0.0012	*P*-value		*p* = 0.0307
Besse [40]	2018	NSCLC	Targeted therapy	NGS	2 weeks (C1D15)	-		-		
Mack [37]	2022	NSCLC	Targeted therapy	NGS	8 weeks	Median PFS	15.1 vs 2.8 mos	Median OS		27.2 vs 15.0 mos
						HR	0.24 (0.13–0.44)	HR		0.30 (0.14–0.66)
						*P*-value	*p* = 0.00001	*P*-value		*p* = 0.003
Iwama [33]	2017	NSCLC	Targeted therapy	NGS	4 weeks	Median PFS	4 wks: 14.3 vs 10.0 mos	-		
						HR	-			
						*P*-value	*p* = 0.52			
Marchetti [32]	2015	NSCLC	Targeted therapy	ddPCR	2 weeks, 2 months	-		-		
Paik [15]	2020	NSCLC	Targeted therapy	NGS	6 weeks, 12 weeks	-		-		
Phallen [26]	2018	NSCLC	Targeted therapy	NGS	Median 19 days	Median PFS	1.6 vs 13.7 mos	-		
						HR	66.6 (13.0–341.7)			
						*P*-value	*p* < 0.0001			
Shaw [23]	2019	NSCLC	Targeted therapy	NGS	6 weeks	Median PFS	6.6 vs 2.6 mos	Median OS	18.0 vs 8.6 mos	
						HR	2.6, 95% Cl: 1.2, 5.8	HR	2.0, 95% Cl: 0.9–4.6	
						*P*-value	-	*P*-value	-	
Shepherd [34]	2018	NSCLC	Targeted therapy	ddPCR	3 or 6 weeks	Median PFS	10.9 vs 5.7 mos	-		
						HR	-			
						*P*-value	-			
Wang [35]	2018	NSCLC	Targeted therapy	ddPCR	8 weeks	Median PFS	11.0 vs 2.1 mos	-		
						HR	-			
						*P*-value	-			
Zhou [14]	2019	NSCLC	Targeted therapy	ddPCR	3 or 6 weeks	Median PFS	19.8 vs 11.3 mos (osi); 10.8 vs 7.0 mos (comp)	-		
						HR	-			
						*P*-value	-			
Bidard [13]	2019	CRC	Targeted therapy	ddPCR	6 weeks	-		Median OS	18 vs 42 mos (for ctDNA (+) vs (-) pre-liver met resection)	
								HR	-	
								*P*-value	*p* < 0.001	
Hong [48]	2016	CRC	Targeted therapy	ddPCR or NGS	2 weeks	-		-		
Kopetz [49]	2020	CRC	Targeted therapy	NGS	1st assessment	-		-		
Nakamura [19]	2021	CRC	Targeted therapy	NGS	3 weeks	Median PFS	5.0 vs 2.2 mos	Median OS	16.5 vs 7.3 mos	
						HR	0.30; 95% Cl, 0.13–0.72	HR	0.31; 95% Cl, 0.12–0.82	
						*P*-value	-	*P*-value	-	
Hrebien [51]	2019	Breast	Targeted therapy	Sequencing & ddPCR	Day 28	Median PFS	Suppressed CDR28 11.1 mos vs. high CDR28 6.4 mos	-		
						HR	0.20 (0.08–0.5)			
						*P*-value	*p* < 0.0001			
Martínez-Sáez [30]	2021	Breast	Targeted therapy	NGS	4 weeks	Median PFS	-	-		
						HR	0.39 (responders, mVAFR <0.3); 0.27 (non-responders, mVAFR >1)			
						*P*-value	*p* = 0.025 (responders, mVAFR <0.3); *p* = 0.010 (non-responders, mVAFR >1)			
Modi [22]	2020	Breast	Targeted therapy	NGS	9 weeks (C4D1)	Median PFS	18.1 vs 6.2 mos	-		
						HR	3.55 (1.53-8.24)			
						*P*-value	*p* = 0.00309			
O’Leary [12]	2018	Breast	Targeted therapy	ddPCR	2 weeks (C1D15)	Median PFS	-	-		
						HR	3.94 (1.61–9.64)			
						*P*-value	*p* = 0.0013			
Ma [52]	2017	Breast	Targeted therapy	NGS	4 weeks	-		-		
Gray [53]	2015	Melanoma	Targeted therapy	ddPCR	4–8 weeks	-		-		
Goodall [55]	2020	mCRPC	Targeted therapy	NGS	8 weeks (C3D1)	Median PFS	-	-		
						HR	2 (1.3–3.2)			
						*P*-value	-			
Jayaram [56]	2021	mCRPC	Targeted therapy	NGS	4 weeks (C2D1)	Varied by gene tested, improved for patients who cleared vs did not clear mutation on-Tx		Varied by gene tested, improved for patients who cleared vs did not clear mutation on-Tx		
Parikh [16]	2020	GI	Targeted therapy	ddPCR	4 weeks	Median PFS	175 vs 59.5 days	-		
						HR	3.29; 95% Cl, 1.55–7.00			
						*P*-value	*p* < 0.0001			
Maron [46]	2020	GE	Targeted therapy	NGS	9 weeks	Median PFS	12.3 vs 3.9 mos (in those with clearance vs not)	-		
						HR	-			
						*P*-value	*p* = 0.02			
Maron [47]	2019	GE	Targeted therapy	NGS	<150 days after metastatic Dx	-		Median OS	13.7 vs 8.6 mos	
								HR	0.3; 95% CI, 0.1–0.8	
								*P*-value	*p* = 0.02	
**Immunotherapy**										
Ricciuti [44]	2021	NSCLC	Immunotherapy	NGS	1st follow up	Median PFS	30.6 vs 3.7 mos	Median OS	NR vs 16.2 mos	
						HR	-	HR	**-**	
						*P*-value	-	*P*-value	**-**	
Cabel [21]	2017	NSCLC, CRC, Uveal mel	Immunotherapy	Bi-PAP, ddPCR or NGS	8 weeks	Median PFS	11 vs 2 mos for ctDNA undetectable vs detectable	Median OS	-	
						HR	-	HR	15 for ctDNA undetectable vs detectable	
						*P*-value	*p* = 0.001	*P*-value	*p* = 0.004	
Zhang [17]	2020	NSCLC, Solid tumors	Immunotherapy	NGS	6–8 weeks	Median PFS	-	Median OS	-	
						HR	0.28 (0.14–0.57) 0.22 (0.04–0.30) 0.3 (0.15–0.60)	HR	0.29 (0.16–0.53) 0.12 (0.04–0.37) 0.29 (0.10, 0.84)	
						*P*-value	-	*P*-value	-	
Assaf [20]	2023	NSCLC	Immunotherapy	NGS	6 weeks	-		Median OS	7.3 mos vs 25.2 mos	
								HR	3.28 (2.2–4.9)	
								*P*-value	*p* < 0.001	
Anagnostou [41]	2019	NSCLC	Immunotherapy	NGS	4-8 weeks	Median PFS	-	Median OS	-	
						HR	-	HR	-	
						*P*-value	*p* = 0.001	*P*-value	*p* = 0.008	
Goldberg [29]	2018	NSCLC	Immunotherapy	NGS	q2 or more weeks	NS due to small sample		Median OS	-	
								HR	0.11 (0.02–0.88) for those who cleared; 0.13 (0.03–0.51) for ctDNA responders	
								*P*-value	*p* = 0.037 (for those who cleared); *p* = 0.0034 (for ctDNA responders)	
Leprieur [43]	2018	NSCLC	Immunotherapy	NGS	2 months	Median PFS	12 mos for pts with ctDNA decrease vs 0.7 mos with ctDNA increase >9%	Median OS	2.2 mos for ctDNA conc >0.006 ng/ul vs NR for conc <0.006 ng/ul	
						HR	-	HR	-	
						*P*-value	*p* < 0.001	*P*-value	-	
Nabet [42]	2020	NSCLC	Immunotherapy	NGS	Various (median 2.4 weeks)	Median PFS	22.4 vs 2.3 mos	-		
						HR	2.28			
						*P*-value	*p* = 0.013			
Thompson [18]	2021	NSCLC	Immunotherapy	NGS	9 weeks	Median PFS	14.1 vs 4.4 mos	Median OS	NR [95% CI lower bound 22.1 mos] v 12.0 mos	
						HR	0.25; 95% Cl, 0.13–0.50	HR	0.27; 95% Cl, 0.12–0.64	
						*P*-value	-	*P*-value	-	
Lee [11]	2018	Melanoma	Immunotherapy	ddPCR	Before week 12	-		Median OS	-	
								HR	wk 6 (*n* = 27) HR for favorable vs unfavorable ctDNA profile: 3.9 (2.1–14.0); wk 12 (*n* = 29) HR: 3.3 (1.0–10.5)	
								*P*-value	*p* = 0.02 (wk 6); *p* = 0.03 (wk 12)	
Lee [54]	2017	Melanoma	Immunotherapy	Not described	Before week 12	Grps A&B sig longer than grp C, but NR; Grp C 2.7 m		Grps A&B sig longer than grp C but NR, grp C 9.8 m		
Dawson [50]	2013	Breast	Immunotherapy	digital PCR or NGS	q3 or more weeks	-		-		
Kim [45]	2018	Gastric	Immunotherapy	NGS	6 weeks	Median PFS	123 vs 66 days	-		
						HR	-			
						*P*-value	*p* = 0.029			
Bratman [58]	2020	Solid tumors	Immunotherapy	NGS	6–7 weeks	Median PFS	-	Median OS	-	
						HR	0.33 (0.19–0.58)	HR	0.36 (0.18–0.71)	
						*P*-value	-	*P*-value	-	

*PFS* progression-free survival, *OS* overall survival, *ctDNA* cell-free circulating tumor DNA, *HR* hazards ratio, *ddPCR* droplet digital PCR, *NSCLC* non-small cell lung cancer, *CRC* colorectal cancer, *mos* months.

Accurate therapeutic response assessment using these methods has also been observed in studies of other targeted therapies in NSCLC. In 51 patients with *ALK* oncogene driven NSCLC receiving various selective EML4-ALK inhibitors, patients with ctDNA clearance using NGS at two months on therapy had better PFS 25.4 vs 11.6 mo, *p* = 0.0012 and OS (NR vs 26.1 mo, *p* = 0.0307) compared to those without ctDNA clearance [38]. Soo and colleagues calculated dVAF of detected variants by NGS at week four in 122 patients enrolled in the CROWN study comparing first-line lorlatinib to crizotinib in *ALK*-positive NSCLC [39]. In both the lorlatinib and crizotinib arms, patients with a complete/partial response or stable disease had a significant decrease in ctDNA compared to baseline. Shaw and colleagues calculated the delta of the mean VAF (dVAF) of *ALK* variants detected by NGS at week six in a cohort of 57 patients receiving second-line lorlatinib therapy [23]. Patients with a complete or partial response had a significant decrease in ctDNA (*p* = 0.0011), while patients with stable or progressive disease did not. In addition, patients with dVAF < 0 had better PFS (HR = 2.6, 95% CI 1.2–5.8) and OS (HR 2.0, 95% CI HR 0.9–4.6) compared to those with dVAF ≥ 0 [23]. In 34 patients with *RET* altered NSCLC treated on the phase 1 trial of selpercatinib, 44% of patients had clearance of the *RET* driver alteration detected by NGS at cycle one (day 15) and 79% had at least a 50% decrease in ctDNA, supporting the clinical activity of the drug [40]. Percent change in *MET* exon 14 skipping driver mutation at six weeks on therapy with tepotinib was assessed in 51 participants of the VISION trial using NGS [15]. In this cohort, 34 patients with a molecular response (defined as a 75-100% decrease in *MET* VAF from baseline) had a complete/partial radiographic response and six patients had stable disease resulting in a disease control rate of 88% [15].

Molecular response assessed by NGS has also demonstrated an association with radiographic response and clinical outcomes in NSCLC patients receiving immune checkpoint inhibitors (ICI) [17, 18, 20, 29, 41–44] A study by Goldberg et al. defined molecular response as a ≥ 50% decrease by the ratio of max VAF on treatment compared to baseline and found that molecular responders had a significantly longer median time on treatment with an ICI (205.5 vs 69 days, *p* < 0.001) and improved PFS (HR 0.29, *p* = 0.03) and OS (HR: 0.13, *p* = 0.007) compared to molecular non-responders) [29]. More recently, Nabet et al. defined molecular response as a ≥ 50% decrease in ctDNA concentration within 4 weeks of treatment initiation in 46 patients receiving ICI monotherapy or with or without a CTLA-4 inhibitor. Patients with a molecular response demonstrated higher radiographic response rates and improved PFS compared to molecular non-responders (22.4 vs 2.3 months, HR 2.28; *p* = 0.013) [42]. In an analysis of 66 patients with advanced NSCLC treated with durvalumab on the ATLANTIC trial, Zhang et al. calculated the ratio of the mean VAF at week six on treatment to the mean VAF at baseline. Patients with a > 50% decrease in VAF were considered molecular responders and had better PFS (HR 0.3, 95% CI 0.15–0.60) and OS (HR 0.29, 95% CI 0.10–0.84) than molecular non-responders [17]. A recent study evaluating serial ctDNA measurements from the phase 3 IMpower150 study demonstrated that changes in ctDNA assessed at week 6 were associated with radiographic response and overall survival [20].

### Molecular response in gastrointestinal cancers

There have been a number of studies published supporting the ability of ctDNA molecular response assessment to predict clinical outcomes in patients with metastatic gastrointestinal cancer treated with immunotherapy or targeted agents (Table 2). In a study of eighteen patients with metastatic gastric cancer that assessed the proportion of ctDNA change using NGS at week six on pembrolizumab, patients with decreasing on-treatment ctDNA levels had improved overall response rates and longer PFS compared to patients without a decrease (ORR 58% versus 0% (*p* = 0.0486), PFS 123 versus 66 days (*p* = 0.029) [4, 45]. Maron et al. similarly showed that patients with *HER2-*positive gastric cancer with clearance of ctDNA assessed by NGS at nine weeks on combination pembrolizumab and trastuzumab had improved PFS compared to those without ctDNA clearance (12.3 months vs 3.9 months, *p* = 0.02) [46]. In another study, in cohort of 35 patients receiving first-line treatment for gastric cancer, those with ≥50% decline in the max VAF (*n* = 23) assessed by NGS had superior OS than those with <50% decline in max VAF (13.7 vs 8.6 months, *p* = 0.02; HR 0.3 95% CI 0.1–0.8) [47].

In 35 patients with *KRAS* mutated metastatic colorectal cancer (mCRC), patients with clearance of the *KRAS* mutation detected by ddPCR at four weeks on treatment with chemotherapy and bevacizumab were found to have improved overall survival compared to those without ctDNA clearance on therapy (42 months vs 18 months, *p* < 0.001) [13]. In 12 patients with *BRAF* V600E mutant mCRC treated with vemurafenib, cetuximab, and irinotecan, clearance or near clearance (>90% decrease) of the *BRAF* mutation detected by ddPCR or NGS at six weeks on therapy correlated with RECIST response [48]. Similarly, in the SWOG S1406 trial of irinotecan and cetuximab with or without vemurafenib in *BRAF* V600E mutant mCRC, 87% of patients in the vemurafenib arm had a reduction in the *BRAF* V600E VAF (percent change) detected by NGS, while none of the patients in the control arm (21% DCR, *P* < 0.001) had a ctDNA decrease. Disease control in the vemurafenib arm was significantly longer compared to the control arm 65% vs 21%, *p* < 0.01), respectively [49]. Parikh et al. studied the percent change in ctDNA VAF by ddPCR at four weeks on chemotherapy and/or targeted therapy in 101 patients with various metastatic gastrointestinal cancers. Patients with a partial response to therapy had a significantly greater percent reduction in ctDNA than those with progressive disease (98% vs 49%, *p* < 0.0001) and patients with >30% decrease in ctDNA had a longer PFS (175 vs 59.5 days; HR 3.29, *p* < 0.0001) [16]. In an analysis of 28 patients enrolled in the phase 2 TRIUMPH trial of pertuzumab plus trastuzumab in *HER2* amplified mCRC, a decrease in the ctDNA fraction at 3 weeks was associated with superior PFS (HR = 0.30; 95% CI, 0.13–0.72) and OS (HR = 0.31; 95% CI, 0.12–0.82) [19].

### Molecular response in breast cancer

A growing number of studies have found an association between molecular response and outcomes in patients with metastatic breast cancer (mBC) treated with various therapies that are either investigational or are now standard of care. In an early proof of concept study involving 30 patients with metastatic breast cancer, ctDNA was detected at baseline in 97% of patients and ctDNA dynamics correlated with changes in tumor burden and outcome [50]. Since that time a number of studies have been published evaluating ctDNA molecular response in breast cancer [12, 30, 51, 52]. In both the PALOMA-3 and BEECH trials of palbociclib plus fulvestrant and capivasertib with or without paclitaxel, respectively, the ratio of on-treatment to baseline *PIK3CA* mutation level was assessed using ddPCR; PALOMA-3 assessed cycle 2 day 1 while BEECH tested multiple time points, determining day 28 was optimal. Both studies found significantly improved PFS in patients with a reduction in *PIK3CA* ctDNA levels on treatment [12, 51]. Recently, ctDNA was analyzed by NGS in 31 mBC patients after one cycle of standard CDK4/6 inhibitor and endocrine therapy. Patients with a mean VAF ratio <0.3 were considered molecular responders and had better PFS (HR 0.39, *p* = 0.025) than molecular non-responders (HR 0.27, *p* = 0.010) [30].

### Molecular response in other solid tumors

In *BRAF* V600 mutated metastatic melanoma, an early study correlated change in ctDNA copies per milliliter at four to eight weeks on treatment with response to MAPK inhibition (vemurafenib, dabrafenib, or dabrafenib/trametinib combination) or immunotherapy (ipilimumab, nivolumab or pembrolizumab) [53]. Eight of ten patients treated with a MAPK inhibitor, responded to therapy and all eight had a 100–1000 fold decrease in ctDNA concentration (*p* = 0.0071). One of the two non-responders also had a 10-fold reduction in ctDNA and had stable disease for >6 months. Conversely, only four of 15 patients treated with immunotherapy responded and there was very little decrease in ctDNA observed in these patients [53]. Two other studies in metastatic melanoma assessed ctDNA clearance or percent change in ctDNA after up to 12 weeks on treatment with immunotherapy, and both demonstrated significantly improved OS among patients with decreasing or cleared ctDNA at the early on-treatment timepoint [11, 54]. A study by Goodall et al. reported on the analysis of ctDNA from 216 patients with metastatic castration-resistant prostate cancer on the A.MARTIN trial of ipatasertib or apitolisib with abiraterone acetate versus abiraterone acetate alone. A reduction in ctDNA by NGS at cycle three day one was associated with improved PFS (HR 2 95% CI 1.3–3.2, *p* < 0.01) and best overall response *p* = 0.024 [55]. More recently, a study Jayaram and colleagues evaluated molecular response in patients with metastatic castration-resistant prostate cancer receiving abiraterone therapy and demonstrated that the clearance of ctDNA assessed by NGS at 3 weeks on therapy was associated with improved overall survival [56].

Multiple studies have also correlated early on-treatment ctDNA changes with response to various immunotherapies across several advanced solid tumors [17, 21, 57, 58]. In 74 patients treated with pembrolizumab as part of the INSPIRE trial, patients with decreasing ctDNA by NGS at cycle three had better response rate (42% vs 2%), PFS (HR 0.33 95% CI 0.19–0.58), and OS (HR 0.36 95% CI 0.18–0.71) [58]. Similarly, samples from 105 patients with various solid tumors from two trials of durvalumab (Study 1108 and Study 10) were analyzed by NGS at baseline and six to eight weeks on therapy. The ratio of the mean VAF < 50% was associated with ORRs and improved PFS (HR 0.28 95% CI 0.25–1.24; HR 0.11 95% CI 0.04–0.30) and OS (HR 0.29 95% CI 0.16–0.53; HR 0.12 95% CI 0.04–0.37) for Study 1108 and Study 10, respectively [17].

### Current limitation and future directions for molecular response

**Fig. 2:**
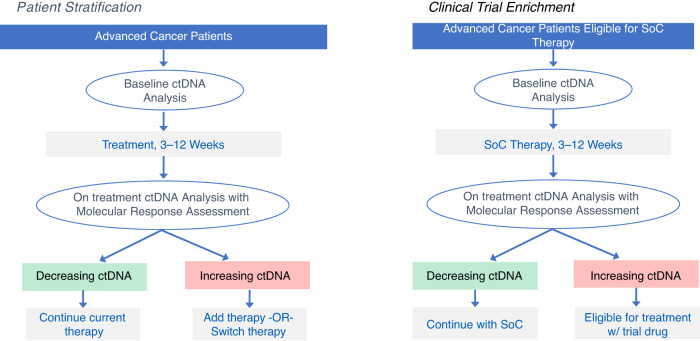
Potential uses of molecular response in the prospective trials. This figure shows two examples of how molecular response (MR) can be incorporated into prospective clinical trials. In the Patient Stratification example, MR is applied to identify a patient population likely experiencing less or little benefit from one treatment regimen earlier than scans and randomize those patients to continue that treatment regimen vs adding or switching therapies. The Clinical Trial Enrichment example, patients with increasing ctDNA early on treatment with standard of care (SOC) are considered eligible for a clinical trial.

Studies published to date highlight the potential use of ctDNA molecular response to predict treatment response and long term outcomes to both targeted agents and immunotherapies across a variety of tumor types. This promising tool may facilitate early re-stratification of patients at high-risk of treatment failure to other effective therapies and at the same time, avoid the potential toxicity of treatment in patients exhibiting a molecular response (Fig. 2). Such an approach will become increasingly important as the number of therapies continues to expand across tumor types and combination treatment strategies become more commonplace. This adaptive approach is already being evaluated in a number of prospective clinical trials (NCT05281406, NCT04093167, NCT04166487). However, the small cohorts and retrospective study design of many studies evaluating molecular response, along with the heterogeneity in optimal early on-treatment timepoints and lack of a consensus definition of molecular response, currently limit the clinical applicability of ctDNA molecular response. Recent studies comparing several of the above approaches to calculating ctDNA molecular response are an important step toward establishing consensus definition of molecular response [17, 18], but additional studies are needed to establish the optimal method and timepoints for calculating molecular response. In addition, the optimal method and specific time-point utilized for calculating ctDNA molecular response may differ according to the specific treatment being evaluated (e.g. targeted therapy vs immune checkpoint inhibitor). Ongoing work by Friends of Cancer Research ctDNA to Monitor Treatment Response (ctMoniTR) project aims to address these questions and further validate these approaches and may help establish the role of ctDNA molecular response in clinical decision-making and as a surrogate endpoint [59]. Another possible avenue for future study would be to assess the impact of combining other established clinical biomarkers of response and resistance with molecular response in predicting therapy response and patient outcomes. We have previously shown that combining single gene negative predictors of response, such as the presence of *STK11* or *KEAP1* mutations, to tumor mutation burden (TMB) improves prediction of response to pembrolizumab [60]. Such an approach could be complementary to molecular response in predicting therapeutic efficacy.

In summary, ctDNA molecular response assessment has been shown to predict outcomes in a number of studies across solid tumors and in patients receiving a broad spectrum of therapeutic strategies. This technology has great potential for use to inform more timely treatment decisions in clinical care and facilitate more efficient clinical trials as a shorter term endpoint, however further validation of the use of molecular response to predict response and outcomes is necessary prior to its integration into clinical practice. Further, prospective trials employing this tool to trigger intervention are needed to establish the clinical utility of molecular response in these scenarios. With these efforts, the promise of ctDNA molecular response as an early on-treatment biomarker may be fully realized.
